# Acute WT1-positive promyelocytic leukemia with hypogranular variant morphology, bcr-3 isoform of PML-RARα and Flt3-ITD mutation: a rare case report: Leucemia promielocítica aguda WT1-positivo com morfologia variante hypogranular, isoforma bcr-3 da PML-RARα e mutação Flt3-ITD: relato de caso raro

**DOI:** 10.1590/1516-3180.2016.020104102016

**Published:** 2017-01-23

**Authors:** Xi Zhang, Cheng Yang, Xiangui Peng, Xinghua Chen, Yimei Feng

**Affiliations:** I MD, PhD. Professor, Department of Hematology, Xinqiao Hospital, Third Military Medical University, Chongqing, China.; II MD. Attending Physician, Department of Hematology, Xinqiao Hospital, Third Military Medical University, Chongqing, China.; III MD. Affiliated Professor, Department of Hematology, Xinqiao Hospital, Third Military Medical University, Chongqing, China.; IV MD, PhD. Full Professor, Department of Hematology, Xinqiao Hospital, Third Military Medical University, Chongqing, China.; V MD, PhD. Assistant Professor, Department of Hematology, Xinqiao Hospital, Third Military Medical University, Chongqing, China.

**Keywords:** Leukemia, promyelocytic, acute, Fms-like tyrosine kinase 3, WT1 proteins, Prognosis, Lung diseases, fungal

## Abstract

**CONTEXT:**

Acute promyelocytic leukemia (APL) accounts for 8% to 10% of cases of acute myeloid leukemia (AML). Remission in cases of high-risk APL is still difficult to achieve, and relapses occur readily.

**CASE REPORT:**

Here, we describe a case of APL with high white blood cell counts in blood tests and hypogranular variant morphology in bone marrow, together with fms-like tyrosine kinase-3 with internal tandem duplication mutations (FLT3-ITD), and bcr-3 isoform of PML-RARα. Most importantly, we detected high level of Wilms’ tumor gene (WT1) in marrow blasts, through the reverse transcription polymerase chain reaction (RT-PCR). To date, no clear conclusions about an association between WT1 expression levels and APL have been reached. This patient successively received a combined treatment regimen consisting of hydroxycarbamide, arsenic trioxide and idarubicin plus cytarabine, which ultimately enabled complete remission. Unfortunately, he subsequently died of sudden massive hemoptysis because of pulmonary infection.

**CONCLUSION:**

Based on our findings and a review of the literature, abnormal functioning of WT1 may be a high-risk factor in cases of APL. Further studies aimed towards evaluating the impact of WT1 expression on the prognosis for APL patients are of interest.

## RESUMO

**CONTEXTO:**

Leucemia promielocítica aguda (LPA) compreende 8% a 10% dos casos de leucemia mieloide aguda (LMA). A remissão em casos de LPA de alto risco ainda é dificilmente conseguida, e recorrência é comum.

**RELATO DE CASO:**

Descrevemos aqui um caso de LPA com glóbulos brancos elevados no exame de sangue e a morfologia variante hipogranular na medula óssea, juntamente com fms-*like* tirosina-quinase-3 com mutações de duplicação em *tandem* interna (FLT3-ITD) e a isoforma bcr-3 de PML- RARα. Mais importante, detectamos alto nível de gene do tumor de Wilms (WT1) em blastos medulares por RT-PCR (*reverse transcription polimerase chain reaction*). Até agora, não há conclusões claras sobre a associação entre os níveis de expressão WT1 e APL. Este paciente recebeu sucessivamente regime de tratamento combinado, de hidroxicarbamida, trióxido de arsênico e idarrubicina e citarabina, alcançando finalmente a remissão completa. Infelizmente, em seguida, ele morreu de repente de hemoptise maciça devido a uma infecção pulmonar.

**CONCLUSÃO:**

Com base em nossos resultados e numa revisão da literatura, a função anormal de WT1 pode ser um fator de alto risco em casos de APL. Novos estudos, com o objetivo de avaliar o impacto da expressão de WT1 no prognóstico dos doentes com APL, são de interesse.

## INTRODUCTION

Currently, with therapeutic improvements that have been attained, curative treatments for acute promyelocytic leukemia (APL) can reach complete response rates close to 90% and long-term relapse-free survival of 85%. However, high white blood cell (WBC) counts are among the high-risk factors for APL, and these proven high risk factors are also seen in subtypes of acute myeloid leukemia (AML). Remission in cases of high-risk APL is still difficult to achieve, and relapses occur readily. Despite major advances in treatments for APL, high-risk APL patients often die during the early treatment because of severe complications. The currently known specific risk factors include fms-like tyrosine kinase-3 (FLT3-ITD), hypogranular variant morphology, and the bcr-3 isoform of PML-RARα.^1^^,^^2^ Here, we describe a case that also expressed high level of Wilms’ tumor gene (WT1), harboring a complex karyotype.

## CASE REPORT

A 42-year-old male patient was hospitalized with hematuria and high white blood cell counts. Physical examination revealed that he had a pale complexion, scattered petechiae and ecchymosis on his skin, and sternal tenderness.

Routine blood tests showed WBC 117.14 × 10^9^/l, hemoglobin (Hb) 68 g/l, platelets (PLT) 23 × 10^9^/l and lactic dehydrogenase (LDH) 320 IU/l. Routine urine tests were positive for urinary protein and urinary red blood cells, and the red blood cell count was 143.6/ul. Among the coagulation parameters, prothrombin time (PT) and activated partial thromboplastin time (APTT) were within the normal range, D2 dimers were elevated to 18 mg/l, and a plasma protamine paracoagulation test was positive. A fecal occult blood test was positive too.

There was no abnormality in pulmonary computed tomography (CT) examination (no fluid and no infection). The sonographic findings from superficial lymph nodes showed no abnormality. Abdominal ultrasound examination showed liver cysts and kidney stones (diameter: 0.7 to 1.0 cm). Cardiac ultrasonography was normal.

**Figure 1:**
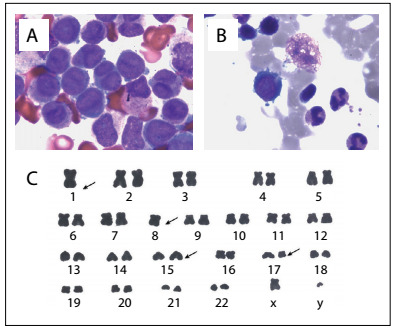
Bone marrow morphology and karyotype analysis: A) Bone marrow smear stained with Wright-Giemsa, showing that this was a rare case of APL with hypogranular variant morphology (M3v). Promyelocytes appeared in the bone marrow aspiration prior to treatment (promyelocytes 91.5%). The cell body was of different size, containing much cytoplasm and few A particles (also known as azurophilic granules). The nuclear distortion was obvious and typically of butterfly or dumbbell shape. B) Complete remission was achieved post-treatment (promyelocytes 1%). C) Karyotype analysis revealed 44,XY, t(15;17)(q22;q21),-1,-8 [1]/ 46,XY, t(15;17) (q22;q21) [6] /46,XY [3]. The clone with the translocation t(15;17)(q22;q21) as the main abnormality was the stem line. One additional abnormal subclone was identified, with loss of a single chromosome on chromosomes 1 and 8 respectively.

**Figure 2:**
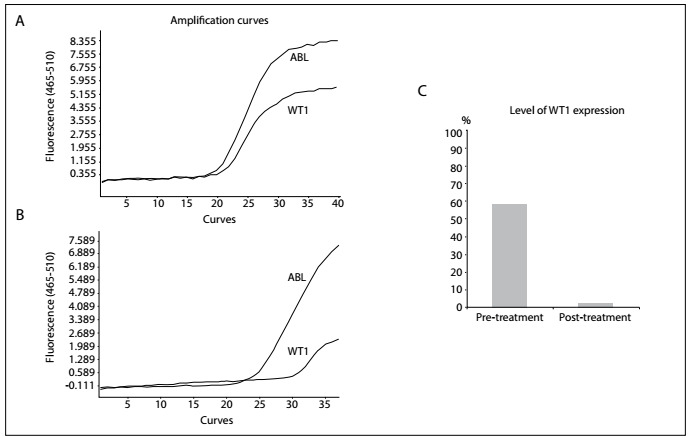
The curves of WT1 genes amplified by means of the real-time polymerase chain reaction (RT-PCR): A) the WT1 value was 641,000 copies at the initial diagnosis and WT1/ABL count of 58.8%; B) the WT1 level reduced to 3630 copies after treatment and WT1/ABL of 2.3%; C) ratio of WT1 to ABL gene before and after treatment.

A bone marrow histological evaluation showed that bone marrow hyperplasia was extremely active. Granulocytes accounted for 99% of the material and, among them, promyelocytes accounted for 91.5%. The cell body was of a different size, containing much cytoplasm and few A particles (also known as azurophilic granules). The nuclear distortion was obvious, typically of butterfly or dumbbell shape. The peroxidase (POX)-positive staining rate was 100%. According to the French-American-British (FAB) criteria, this case was classified as APL with hypogranular variant morphology (M3v) (Figure 1). A peripheral blood smear indicated that myeloblasts accounted for 6% and promyelocytes accounted for 92%, and the morphology was similar to what was seen in the bone marrow smear. Flow cytometry detected that the abnormal bone marrow cell population accounted for 93.6%. These abnormal bone marrow cells were positive for CD45, CD117, CD13/CD33, CD64, CD38, MPO, CD34, HLA-DR, CD2, CD4 and CD19 expression, which suggested that this case consisted of AML (B cells^+^ and T cells^+^). Fluorescence *in situ* by hybridization (FISH) detection showed that the fusion gene PML/RARα accounted for 94%. PCR experiments were bcr-3 subtype PML/RARα-positive and FLT3-ITD-positive and the WT1 count was 58.8% (Figure 2).

The chromosome analysis displayed: 44,XY, t(15;17)(q22;q21), -1,-8 [1]/ 46,XY, t(15;17)(q22;q21) [6]/46,XY [3]. Apart from the typical translocation t(15; 17)(q22;q21), which was the main abnormality in APL, one additional abnormal subclone consisted of loss of a single chromosome on chromosomes 1 and 8 respectively (Figure 1). This patient was diagnosed as presenting APL with hypogranular variant morphology (M3v), short (bcr-3) subtype and FLT3-ITD mutation, and was WT1-positive.

**Figure 3:**
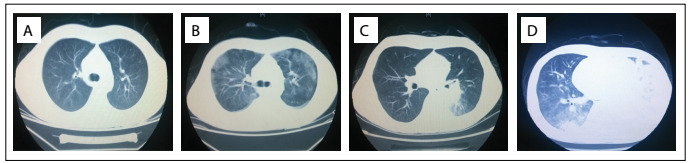
Chest computed tomography scans: A) There were no abnormal changes in the lungs prior to treatment (no pleural effusion and no infection); B) With the promyelocyte differentiation syndrome, computed tomography (CT) scans of the patient’s chest showed ground-glass opacity (GGO) in the two lung lobes and pleural effusion; C) After hormonal therapy, CT showed clear reduction of bilateral pleural effusion, such that the right side of the pleural effusion was basically absorbed. However, the lung consolidation with halo signs in the left pulmonary hilum means that fungal infection was likely; D) Massive pleural effusion was seen in the left lung, giving rise to pulmonary atelectasis, with patchy change in the right lung, when there was complete remission from acute promyelocytic leukemia (APL).

The initial treatment for this patient comprised hydroxycarbamide, low-dose cytarabine and arsenic trioxide (ATO; 10 mg/d). To avoid retinoic acid syndrome (RAS), all-trans retinoic acid was not administered at first, during the high white blood cell phase. At the same time, we improved the anemia through transfusion of red blood cells, corrected the abnormal coagulation through plasma transfusion and implemented other symptomatic supportive treatment. After four days of ATO administration, the patient complained of fever (39 °C), chest tightness and shortness of breath. An ultrasound examination showed a small amount of pleural effusion and pericardial effusion. Considering these as side effects of arsenite, we stopped using it and replaced it with idarubicin plus cytarabine/chemotherapy regimen (IA). When IA chemotherapy ended, this patient had developed dyspnea, and blood gas analysis showed type I respiratory failure (pH 7.55; PO_2_ 59 mmHg). A pulmonary spiral computed tomography (CT) examination showed frosted glass-like changes to the lung fields and medium amounts of pleural effusion (Figure 3). Because of the presence of promyelocyte differentiation syndrome, the patient was placed under continuous oxygen therapy, with 80 mg/day of methylprednisolone.

After ten days of hormonal therapy, the symptoms of heart fatigue and shortness of breath showed clear improvement. CT on the lungs showed that a clear reduction of bilateral pleural effusion had occurred and that the right-side pleural effusion had basically been absorbed. One high-density patchy shadow (of dimensions 4.4 x 3.5 cm) was discovered in the left pulmonary hilar (Figure 3).

The patient then entered a bone marrow suppression period and contracted repeated high fever. Considering the possibility of fungal infection, we replaced the hormone treatment with voriconazole, based on broad-spectrum antibiotics. On the 17^th^ day after IA chemotherapy ended, the patient still repeatedly presented high fever. Routine blood tests showed WBC 5.34 × 10^9^/l, Hb 62 g/l and PLT 37 × 10^9^/l, Pulmonary CT reexamination showed significant consolidation of lung tissue in the left lower lung lobe, and that the high-density mass had increased to a size of 4 .7 x 3.5 cm. No obvious evidence of tuberculosis, bacteria or fungi was found through pleural fluid drainage examination.

On the 26^th^ day after the IA regimen ended, a sputum culture experiment showed the presence of mucosal infection. We replaced the voriconazole with posaconazole for patient and, at the same time, instituted oral retinoic acid therapy.

On the 44^th^ day after chemotherapy, routine blood reexamination showed WBC 5.84 × 10^9^/l, Hb 73 g/l and PLT 404 × 10^9^/l. The morphology of the bone marrow presented complete remission (Figure 1). Pulmonary CT reexamination showed massive pleural effusion in the left lung, giving rise to total pulmonary atelectasis (Figure 3). In contrast, the right-side pleural effusion was completely absorbed. A tuberculosis antibody test was positive in pleural fluid and blood samples, and investigation of fungus and promyelocytes in pleural fluid was negative. Implementation of diagnostic anti-tuberculosis therapy was then planned. However, the patient suddenly died on the third day after tuberculosis antibody detection, because of massive hemoptysis.

## DISCUSSION

APL is a distinctive subtype of myeloid malignancies, characterized by reciprocal translocation between chromosomes 15 and 17. This generates three kinds of PML-RARα fusion genes, referred to as long (L or bcr-1), variant (V or bcr-2) and short (S or bcr-3). The first-line treatment for newly diagnosed APL has mainly been based on a combination of all-trans-retinoic acid (ATRA) and anthracycline drugs, which has achieved good effects.^3^^,^^4^

In this paper, we presented one case of the bcr-3 subtype of APL. According to the literature, the efficacy of type bcr-3 is often worse than that of type bcr-1, and patients with bcr-3 tended to have more relapses and shorter survival.^2^ In an *in vitro* experiment, in the absence of granulocyte-macrophage colony-stimulating factor (GM-CSF), bcr-3 cells had anti-apoptotic properties and ATRA inhibited the growth of bcr-1 cells more strongly than bcr-3 cells, which suggested that patients with bcr-3 APL may have stronger drug resistance to ATRA.^5^ This can be contrasted with cases of retinoic acid syndrome: our patient with high white blood cell counts did not receive ATRA during the first-course treatment.

Some researchers have believed that there is a high-degree correlation between the bcr-3 subtype and FLT3 mutations. Additionally, FLT3 mutations are often associated with high leukocyte states, which are one of the important adverse prognostic markers of APL.^6^^,^^7^ FLT3 mutations mainly consist of internal tandem repeat (ITD) and tyrosine kinase domain (TKD) mutations. Several reports have mentioned that there is a close association between FLT3-ITD and elevated white blood cell counts, hypogranular variant morphology (M3v) and the bcr-3 isoform of PML-RARα, which is consistent with our report. FLT3-ITD mutation activates tyrosine kinase and downstream signaling pathways such as STAT5, RAS/MAPK and PI3K/AKT, ultimately leading to inhibition of cell apoptosis, but it accelerates excessive proliferation, which results in high white blood cell counts. A high frequency of FLT3-ITD was previously reported in 30-45% of APL patients. The FLT3-ITD mutation of APL had a lower remission rate and shorter overall survival phase.^8^^,^^9^

Apart from the FLT-ITD mutation, WT1 expression was also seen in the case of APL that we report here. Overexpression, polymorphisms and mutations of the WT1 gene have been reported in AML and have variably been correlated with the prognosis. Moreover, FLT3 mutations were found in 37% of APL patients and correlated with high WT1 mRNA expression.^10^ Recent studies on AML patients have shown that high WT1 expression was specifically correlated with presence of the FLT3-ITD mutation.^10^^,^^11^^,^^12^WT1 mutation is currently recognized as an adverse prognostic factor for AML,^13^^,^^14^ but information on its impact on the prognosis for APL is lacking. In 2012, Gaur et al.^10^ published the first analysis of WT1 gene variations focusing on APL. WT1 mutations were detected in four of the 103 patients with APL, but they found no differences in WT1 expression levels between patients classified as low, intermediate or high risk according to the Sanz score. Hecht et al.^15^ found that patients with high WT1 expression achieved a complete remission (CR) significantly faster. There was no difference in the cumulative incidence of relapse and the time until relapse between different WT1 expression groups. Lastly, no clear conclusions about the association between WT1 expression levels and the cause of shorter overall survival (OS) after CR can be drawn. However, the risk of death after CR was nine times higher in the low WT1 group and 10.5 times higher in the high WT1 group than among patients with intermediate WT1 expression. Furthermore, in univariate analysis, high WT1 was also a predictor of shorter relapse-free survival (RFS).^15^ Based on our result, the WT1 value was 641,000 copies at the initial diagnosis. When the disease was in complete remission, the polymerase chain reaction (PCR) reexamination showed that the WT1 level reduced to 3630 copies. The ratio of WT1 to the ABL (abelson tyrosine-protein kinase) decreased from 58.8% to 2.3% (Figure 2).

In conclusion, there is controversy as to whether high WT1 expression suggests a poor prognosis for AML. However, we believe that high WT1 expression in APL cases indicates an adverse prognosis and should be considered in APL risk stratification. Although the clinical data for further validation is far from plentiful, WT1 inhibitor or WT1-specific cytotoxic cell therapy may be promising in cases of high-risk APL with WT1 overexpression.

We reviewed the literature in MEDLINE, PubMed, Embase and LILACS using the English keywords “acute promyelocytic leukemia”, “FLT3-ITD” and “WT1”. We found that only two WT1-positive APL patients with FLT3-ITD mutation and bcr-3 isoform PML-RARa expression had previously been reported. Both of them died during early treatment.^16^ In addition, Zou et al.^17^ and Liu et al.,^18^ reported two cases of APL with FLT3-TKD mutation that were WT1-positive, and both of these patients suffered systemic relapse. However, neither the bcr subtype of PML-RARα nor the specific cell morphology was mentioned (Table 1).

**Table 1:**
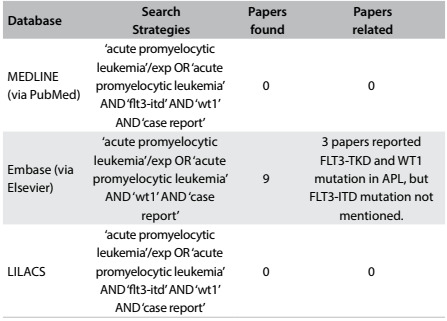
Database search results for acute promyelocytic leukemia, FLT3-ITD and WT1 on August 5, 2016

Our case was similar to that of Greco et al.^16^ Moreover, our patient presented a complex karyotype through chromosome analysis and mixed molecular expression according to flow cytometry. After treatment, our patient achieved complete remission, but unfortunately he died of pulmonary infection. The direct cause of death was massive hemoptysis, probably caused by tuberculosis or by fungus eroding blood vessels.

Although the treatment for the pulmonary infection was sufficient and timely, our patient died because of symptoms as mentioned above. Through tracing these symptoms back to their sources, it could be seen that the previous chemotherapy and glucocorticoid applications may have led to serious infection in this patient. This is also a lesson that doctors need to be aware of, regarding the possibility of infection in high-risk APL patients during induction therapy. Although diagnosing and treating high-risk APL are very important, we also learned that severe complications, such as disseminated intravascular coagulation, hydrothorax and serious infection, which may occur especially in cases of high-risk APL, need to be treated as soon as possible. While reducing the tumor burden of APL, lack of care regarding these complications might be fatal.

## CONCLUSION

We presented a rare case of APL with hypogranular variant morphology, bcr-3 isoform of PML-RARα and Flt3-ITD mutation, which was also WT1-positive. Based on our findings and a review of the literature, abnormal functioning of WT1 may be a high-risk factor for APL. Further studies aiming to evaluate the impact of WT1 expression on the prognosis of APL patients are of interest.
